# RNA Modifications in T cell Immunity: Mechanisms, Disease Relevance, and Therapeutic Potential

**DOI:** 10.7150/thno.124482

**Published:** 2026-01-01

**Authors:** Xinyuan Zhao, Meiyan Zou, Weiyao Feng, Nina Li, Xu Chen, Pei Lin, Zihao Zhou, Yunfan Lin, Li Cui

**Affiliations:** Stomatological Hospital, School of Stomatology, Southern Medical University, Guangzhou, 510280, Guangdong, China.

**Keywords:** RNA modifications, T cell immunity, therapeutic potential, epitranscriptomics, immune regulation

## Abstract

RNA modifications constitute a versatile and dynamic layer of post-transcriptional regulation that enables T lymphocytes to fine-tune gene expression programs in response to developmental, environmental, and pathogenic cues. Chemical marks such as N^6^-methyladenosine (m^6^A), 5-methylcytosine (m^5^C), and pseudouridine (Ψ) shape transcript stability, splicing, localization, and translation through coordinated actions of writer, reader, and eraser proteins. Emerging evidence reveals that these pathways orchestrate T cell lineage specification, activation thresholds, effector-memory balance, and immune tolerance, while their dysregulation contributes to infection, autoimmunity, malignancy, and graft rejection. Integrating findings across m^6^A and other epitranscriptomic marks—including m^5^C, Ψ, N^7^-methylguanosine (m^7^G), N^1^-methyladenosine (m^1^A), N^4^-acetylcytidine (ac^4^C), and N^6^-2'-O-methyladenosine (m^6^A_m_) —this review delineates how distinct RNA modifications converge on shared molecular circuits controlling transcriptional, metabolic, and signaling networks in T cell immunity. Aberrant modification patterns reshape cytokine profiles, mitochondrial metabolism, and antigen-driven responses, thereby influencing disease trajectories across diverse pathological contexts. Collectively, these insights establish RNA modification as a central regulatory axis linking transcriptomic plasticity to immune function and therapeutic responsiveness. We further highlight unresolved challenges—such as defining spatiotemporal modification landscapes and achieving selective pharmacological modulation—and propose integrative multi-omics and *in vivo* perturbation approaches to translate epitranscriptomic mechanisms into targeted immunotherapies.

## Introduction

T lymphocytes constitute the cornerstone of adaptive immunity, orchestrating antigen-specific recognition, clonal expansion, and effector responses that collectively ensure the elimination of infected or malignant cells while maintaining immune tolerance and tissue homeostasis 1-3. Their capacity to mount precise and flexible immune responses relies on the coordinated interplay of multiple regulatory layers, encompassing transcriptional, epigenetic, post-transcriptional, and metabolic control 4-6. These mechanisms operate in an integrated and temporally synchronized manner to align T cell activation, differentiation, and resolution of effector responses with immunological demands. Disruption of this multilayered regulation can result in either immune insufficiency or immunopathology, underscoring the necessity for precise control mechanisms that enable T cells to dynamically adapt to diverse environmental and pathogenic cues 7, 8.

Among these regulatory dimensions, post-transcriptional control is particularly crucial. Following antigen encounter, T cells must rapidly remodel their transcriptome and proteome to sustain proliferation, metabolic reprogramming, and effector molecule production 9-11. Unlike transcriptional regulation, which requires new gene activation, post-transcriptional processes allow immediate adjustment of existing transcripts, conferring temporal precision and energetic efficiency. RNA modifications have recently emerged as a central post-transcriptional mechanism that adds a chemical layer of information to RNA molecules 12. Through covalent modification of messenger RNAs, transfer RNAs, ribosomal RNAs, and non-coding RNAs, chemical marks such as N^6^-methyladenosine (m^6^A), 5-methylcytosine (m^5^C), pseudouridine (Ψ), N^7^-methylguanosine (m^7^G), N^1^-methyladenosine (m^1^A), and N^4^-acetylcytidine (ac^4^C) dynamically regulate RNA stability, splicing, export, localization, and translation 13. These modifications are installed, recognized, and removed by dedicated “writer”, “reader”, and “eraser” proteins that collectively integrate upstream signaling events with downstream gene expression outcomes 14, 15.

Mounting evidence indicates that these RNA modification pathways act as key modulators of T cell fate and function 16. By controlling the stability and translation of lineage-defining transcription factors, signaling mediators, and metabolic enzymes, RNA modifications influence T cell differentiation, activation thresholds, effector-memory transitions, and regulatory T cell stability 17-22. They serve as molecular interfaces that couple cytokine and metabolic signals to transcriptional and functional reprogramming, ensuring the precise tuning of immune responses. Conversely, perturbations in RNA modification machinery—whether through altered enzyme expression, localization, or substrate availability—can rewire T cell programs, promoting immune dysregulation, chronic inflammation, or tumor immune evasion 10. These findings position the epitranscriptome as a pivotal integrator that links transcriptional, metabolic, and signaling networks to T cell functionality and immune homeostasis.

Despite substantial advances, critical gaps remain in understanding how distinct RNA modifications are coordinated to regulate T cell biology. The temporal and spatial dynamics of modification deposition during T cell activation and differentiation remain incompletely mapped. Moreover, the functional interplay among different modification types—such as m^6^A, m^5^C, and Ψ—and their crosstalk with chromatin remodeling and non-coding RNA networks are only beginning to be explored. Technical limitations in detecting low-abundance modifications within small immune subsets, coupled with the challenge of causally linking modification events to specific functional outcomes *in vivo*, continue to constrain mechanistic insight. A comprehensive and integrative framework is therefore needed to elucidate how RNA modifications orchestrate the molecular circuitry underlying T cell immunity. Deciphering this interplay will deepen our understanding of immune regulation and open new therapeutic avenues. Selective targeting of RNA modification enzymes or pathways holds promise for reprogramming immune responses in autoimmunity, infection, cancer, and transplantation, representing an emerging frontier for precision immunotherapy.

In this work, we integrate current knowledge across three interrelated dimensions that collectively distinguish it from previous publications. First, we provide a comprehensive synthesis of multiple RNA modification types—including m^6^A, m^5^C, Ψ, m^7^G, m^1^A, ac^4^C, and N^6^-2'-O-methyladenosine (m^6^A_m_) —in the context of T cell biology, rather than focusing on a single modification or immune cell subset as in prior reviews. Second, we systematically examine how these epitranscriptomic mechanisms shape diverse disease landscapes, extending beyond cancer and autoimmunity to encompass viral and bacterial infections, cardiovascular disease, and transplant immunology. Third, we emphasize mechanistic depth, detailing how RNA modifications regulate signaling pathways, transcriptional networks, and metabolic programs underlying T cell activation, lineage specification, and immune tolerance. Finally, we outline future challenges and directions, such as spatiotemporal mapping and therapeutic translation, thereby establishing a conceptual and technological framework that advances the field beyond existing summaries of m^6^A-centric regulation.

## RNA modifications: chemical principles, analytical strategies, and functional implications

### Chemical basis and regulatory mechanisms of RNA modifications

RNA modifications constitute a dynamic post-transcriptional layer of gene regulation that fine-tunes RNA stability, translation, and localization 15. More than 170 distinct chemical marks have been identified 23, among which m^6^A 24, m^5^C 25, Ψ 26, m^7^G 27, m^1^A 28, and ac^4^C 29 are the most functionally characterized. These modifications occur at defined sequence motifs or structural contexts and are catalyzed by dedicated enzymatic machineries commonly referred to as writers, erasers, and readers 30, 31.

The m^6^A writer complex, composed of METTL3, METTL14, and WTAP, acts co-transcriptionally on DRACH motifs to control mRNA turnover and translation 32-34. Accessory factors such as VIRMA, RBM15/15B, and ZC3H13 regulate substrate selectivity and subcellular deposition 35-38. In T cells, METTL3-mediated methylation promotes clonal expansion and effector differentiation, whereas its loss impairs memory maintenance and cytokine expression 17. Reversibility is provided by erasers—FTO and ALKBH5—which demethylate m^6^A-marked transcripts to modulate apoptosis and metabolic adaptation 39, 40. Reader proteins such as YTHDF1/2/3 and YTHDC1/2 interpret these marks to determine RNA stability, translation efficiency, and localization, thereby coupling post-transcriptional control to T cell activation thresholds 41-47.

Beyond m^6^A, other modification machineries participate in immune regulation. The NSUN family installs m^5^C marks that enhance translational fidelity and mitochondrial homeostasis 48-54, while DNMT2 methylates tRNA^Asp^ to sustain protein synthesis under stress 55. Ψ synthases (PUS10) and the acetyltransferase NAT10 introduce Ψ and ac^4^C modifications, respectively, to stabilize RNA conformation and improve decoding accuracy 56, 57. The m^7^G cap is installed by the RNMT-RAM complex, which is essential for mRNA stability, nuclear export, and efficient translation initiation 58. The TRMT6/61A complex catalyzes m^1^A modification primarily at position 58 of tRNAs, maintaining translational fidelity and tRNA stability 59. Importantly, these enzymatic systems are not static; their abundance and activity are governed by metabolic and signaling pathways—such as mTOR, HIF-1α, and cytokine-driven JAK/STAT cascades—linking extracellular cues to post-transcriptional outputs that orchestrate T cell activation, differentiation, and exhaustion 60-62. Together, these interdependent machineries establish a multilayered regulatory hierarchy through which diverse RNA marks confer transcriptional flexibility, metabolic adaptability, and immune precision upon T cells.

### Technologies and analytical approaches for RNA modifications

Technological advances in biochemical and sequencing methodologies have revolutionized the detection and quantification of RNA modifications at transcriptome-wide and even single-molecule resolution. Conventional mapping strategies fall into three main categories: chemical or enzymatic probing, immunoprecipitation-based enrichment, and direct detection using nanopore or single-molecule real-time sequencing 63. Among these, nanopore direct RNA sequencing (DRS) has emerged as a powerful approach that enables simultaneous identification of multiple modification types without chemical pretreatment, offering quantitative insights into modification stoichiometry and transcript distribution 64. Computational frameworks complement these advances by integrating raw signal features, sequence context, and biological replicates to distinguish true modification events from background noise 65. Deep learning-based algorithms such as modCNet further improve precision, allowing concurrent identification of multiple cytidine modifications, including m^5^C and ac^4^C, from a single sample 66. These developments expand the analytical capacity to dissect complex modification networks that coordinate RNA stability, translation, and metabolism. Although direct single-cell mapping of RNA modifications remains technically challenging, integration of modification profiling with single-cell transcriptomic and spatial analysis has begun to reveal how RNA modification enzyme expression and activity vary across T cell states and tissue niches. Such combined approaches promise to elucidate how post-transcriptional regulation contributes to T cell activation, differentiation, and exhaustion within diverse immune microenvironments.

### Biological significance and functional consequences of RNA modifications in T cells

RNA modifications form an essential post-transcriptional regulatory layer that enables T cells to fine-tune gene expression programs in response to environmental and metabolic cues 67, 68. By introducing reversible chemical marks onto coding and non-coding RNAs, these pathways control transcript stability, splicing, export, and translation, allowing T cells to rapidly transition between quiescent, activated, and memory states 69. This dynamic adaptability ensures that immune responses are both flexible and precisely timed without requiring complete transcriptional reprogramming. Functionally, the epitranscriptomic machinery establishes a coordinated network that balances activation, persistence, and exhaustion. Individual RNA modifications do not act in isolation but instead intersect to modulate shared molecular circuits governing transcriptional, metabolic, and signaling pathways 67, 70. By integrating these post-transcriptional checkpoints, T cells dynamically adjust to antigenic load, nutrient fluctuations, and cytokine gradients, maintaining effector potential while preventing overstimulation or premature dysfunction.

Recent discoveries highlight that aberrant RNA modification programs can profoundly influence T cell fate and immune homeostasis. Elevated METTL16-mediated m^6^A deposition increases methylation of *TCF-1* mRNA, limiting TCF-1 stability and constraining the pool of self-renewing precursors essential for sustained cytotoxic responses. Loss of METTL16 restores TCF-1-driven stem-like properties, improving chimeric antigen receptor (CAR)-T cell persistence and antitumor efficacy 71. Conversely, excessive METTL3-dependent m^6^A methylation stabilizes inhibitory receptor transcripts and suppresses cytokine production, reinforcing T cell exhaustion under chronic stimulation. Inhibition of METTL3 reverses these effects and reactivates effector function 72. Beyond m^6^A, additional RNA modification systems exert equally critical control over T cell biology. ac^4^C modification of mRNA by NAT10 enhances translational efficiency of *MYC* transcripts upon activation, driving cell cycle progression and clonal expansion. Loss of NAT10 impairs proliferation and antiviral immunity, and its age-associated decline contributes to reduced immune responsiveness, positioning RNA acetylation as a determinant of T cell vigor 73. In parallel, NSUN4-mediated m^5^C modification promotes CD8^+^ T cell exhaustion in systemic lupus erythematosus (SLE) by elevating CD74 expression and activating CD44-mTOR-dependent mitophagy. Silencing NSUN4 with siRNA-loaded nanoparticles restores mitochondrial integrity and cytotoxic capacity, underscoring m^5^C methylation as a key post-transcriptional regulator of immune dysfunction 60. Collectively, these insights define RNA modifications as integral mediators of T cell function that couple environmental sensing with metabolic and transcriptional programs. Their context-dependent and reversible nature endows T cells with functional precision yet also creates vulnerabilities that can be therapeutically exploited. Selective modulation of RNA-modifying enzymes therefore represents a promising approach to reprogram immune cell states and restore immune competence across cancer, infection, and autoimmune disease.

## Epitranscriptomic regulation of T cell development and function

RNA modifications, particularly m^6^A, m^5^C, Ψ, and other chemical marks, have emerged as pivotal post-transcriptional regulators of T cell fate, effector function, and immune homeostasis 74-77. These modifications influence mRNA stability, splicing, translation, and decay, thereby integrating environmental cues, metabolic status, and activation signals into precise gene-expression programs 78. Accumulating evidence from distinct T cell subsets reveals that RNA modification enzymes and readers orchestrate immune responses through highly context-dependent mechanisms 79, 80.

### m^6^A modification in CD8^+^ T cell differentiation and survival

In the cytotoxic T cell lineage, m^6^A methylation serves as a pivotal epitranscriptomic regulator that orchestrates both effector differentiation and memory maintenance 81, 82. The methyltransferase METTL3 is essential for the expansion and lineage progression of CD8^+^ T cells during acute viral infection. Conditional Mettl3 deletion impairs effector proliferation and terminal differentiation, consequently compromising memory formation and secondary responses. Integrative RNA-seq and m^6^A-miCLIP-seq analyses demonstrate that METTL3 modulates a network of cell-cycle and transcriptional regulators, including stabilizing *Tbx21* mRNA to sustain T-bet expression and effector programming. Ectopic T-bet expression partially restores differentiation defects, underscoring a direct mechanistic link between m^6^A-mediated mRNA stabilization and lineage-specifying transcriptional circuits 17. In parallel, m^6^A demethylation provides a counter-regulatory mechanism that preserves cytotoxic T cell survival. The demethylase FTO fine-tunes activation-induced apoptosis by modulating m^6^A methylation on *Fas* transcripts. Loss of FTO leads to excessive methylation and IGF2BP3-dependent stabilization of *Fas* mRNA, resulting in upregulated Fas expression and enhanced apoptosis in activated CD8^+^ T cells. Disruption of the Fas m^6^A sites or IGF2BP3 knockdown restores normal Fas turnover and rescues T cell viability 79. Collectively, these findings delineate an integrated post-transcriptional program in which m^6^A dynamically couples transcriptional reprogramming, cell-cycle progression, and apoptotic control to coordinate CD8^+^ T cell fate decisions. By adjusting the stability and translation of lineage-defining and pro-survival transcripts, m^6^A ensures the precise balance between effector expansion and memory persistence, providing a mechanistic framework for epitranscriptomic regulation of cytotoxic immunity.

### m^6^A-dependent post-transcriptional regulation of CD4^+^ T cell activation and effector function

Building on the regulatory logic established in cytotoxic T cells, m^6^A methylation exerts multifaceted control over CD4^+^ T cell activation and lineage specialization by coordinating cytokine expression, co-stimulatory signaling, and transcriptional programming 83, 84. In CD4^+^ T lymphocytes, m^6^A dynamically shapes effector responses through selective regulation of transcript stability. Upon activation, *Tnf* mRNA acquires increased m^6^A deposition within its 3′ untranslated region, enabling recognition by the reader protein YTHDF2, which accelerates mRNA decay and constrains TNF production. This post-transcriptional mechanism links RNA methylation to the resolution of inflammatory signaling, preventing excessive cytokine output while sustaining appropriate effector activity 80. m^6^A methylation also governs the expression of CD40L, a co-stimulatory molecule essential for CD4^+^ T cell activation and immune coordination. The antagonistic actions of METTL3 and FTO modulate the methylation status of *Cd40l* transcripts, whereas YTHDF2 promotes degradation of methylated mRNAs. This regulatory circuit fine-tunes CD40L expression and downstream cytokine programs, thereby determining the amplitude and qualitative polarization of CD4^+^ T cell responses 85. In specialized helper subsets, METTL3-catalyzed m^6^A deposition preserves the stability of lineage-defining transcripts and maintains the transcriptional network of T follicular helper (Tfh) cells. Loss of METTL3 in CD4^+^ T cells impairs germinal-center formation and reduces expression of Tcf7, Bcl6, Icos, and Cxcr5 in a methyltransferase activity-dependent manner. m^6^A deposition within the* Tcf7* 3′UTR stabilizes the transcript, whereas its removal accelerates decay and disrupts TCF-1-driven gene programs. Restoration of TCF-1 rescues these defects, directly linking m^6^A-dependent RNA stabilization to the maintenance of helper-cell identity and humoral immunity 86. Within the regulatory lineage, METTL14 expression increases during the induction of induced regulatory T cells (iTregs) from naïve CD4^+^ precursors, where it sustains FOXP3 expression and suppressive function. Silencing METTL14 destabilizes *Foxp3* mRNA, impairs iTreg differentiation, and enhances expression of pro-inflammatory cytokines such as IFN-γ and IL-17A, leading to compromised immunosuppression *in vitro* and in colitis models. Mechanistically, METTL14 deficiency activates the mTOR-p70S6K signaling cascade, a pathway known to undermine iTreg stability and function 87. Collectively, these findings reveal m^6^A methylation as a key post-transcriptional mechanism that integrates signaling intensity, transcriptional programming, and metabolic state to coordinate CD4^+^ T cell activation and lineage differentiation. Through the concerted actions of methyltransferases, demethylases, and reader proteins, m^6^A dynamically governs the stability and translation of transcripts that define effector, helper, and regulatory fates. This integrated framework underscores the central role of RNA methylation in maintaining CD4^+^ T cell functional balance and highlights its pathological relevance in autoimmunity, chronic inflammation, and tumor immunity.

### m^6^A-mediated specification of γδ T and iNKT cell lineages

Extending beyond conventional αβ T cells, m^6^A methylation also governs unconventional T cell subsets that bridge innate and adaptive immunity 88. γδ T cells rapidly respond to stress signals and maintain epithelial integrity, whereas invariant natural killer T (iNKT) cells recognize lipid antigens presented by CD1d and exert potent immunoregulatory and antitumour functions through immediate cytokine secretion 89. These lineages rely on distinct transcriptional programs and thymic developmental cues, both of which are subject to post-transcriptional regulation by m^6^A.

Loss of the RNA demethylase ALKBH5 in lymphocytes results in a pronounced expansion of γδ T cells, enhancing mucosal defence against Salmonella typhimurium. In thymocytes, ALKBH5 deficiency elevates global m^6^A levels and represses critical Notch pathway components—including Jagged1 and Notch2—thereby attenuating Notch signalling. The resulting transcriptional shift promotes γδ T cell precursor proliferation and differentiation, expanding the mature γδ T cell compartment 90. In addition, METTL3-dependent methylation fine-tunes γδ T cell subset balance by regulating mRNA stability and endogenous double-stranded RNA (dsRNA) levels. By promoting *Stat1* mRNA degradation and preventing dsRNA accumulation, m^6^A methylation limits aberrant STAT1 activation, thereby sustaining γδ T17 differentiation and IL-17 production 91.

**Figure 1:**
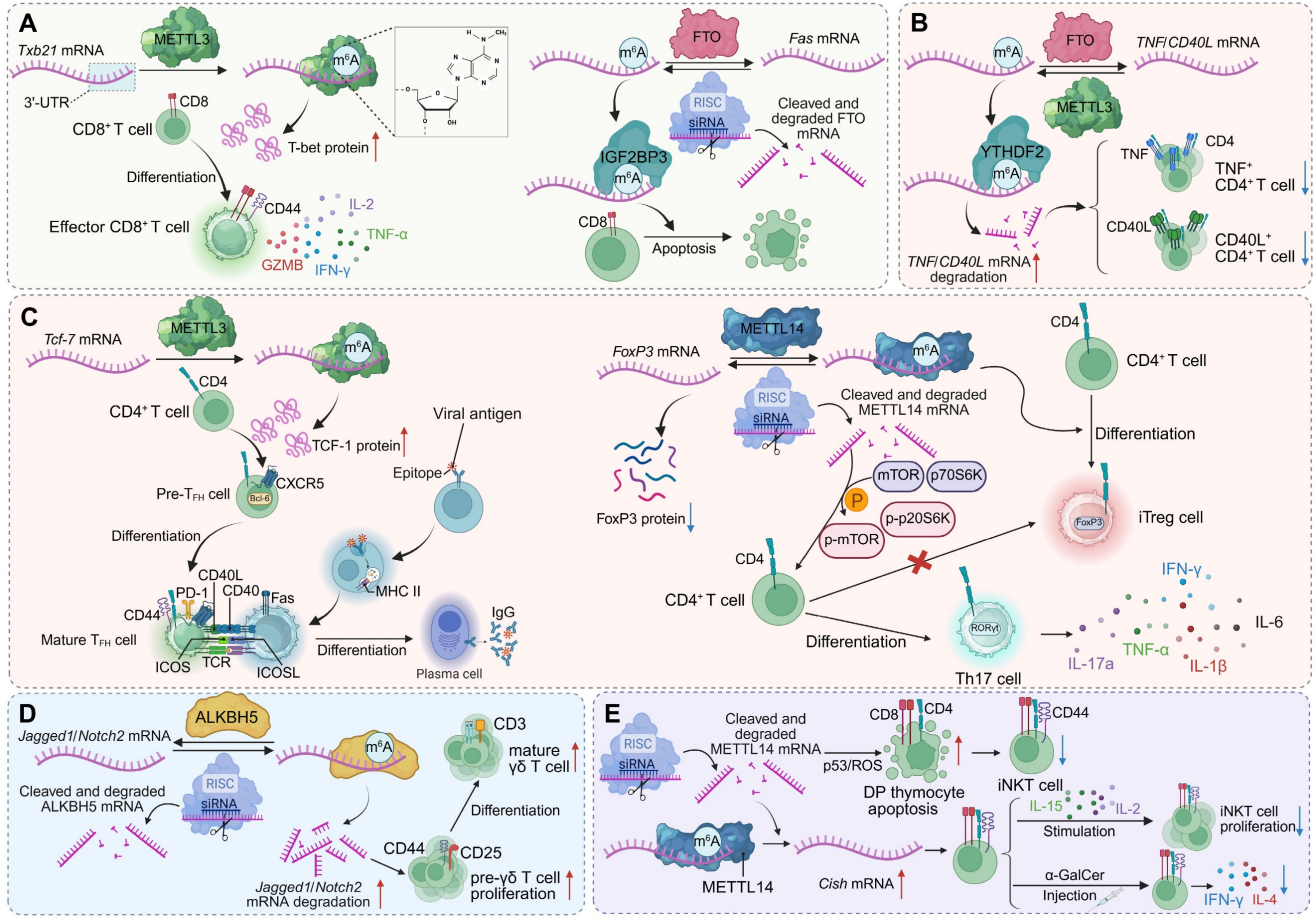
** m^6^A modification regulates T cell lineage commitment and effector functions. (A)** METTL3-mediated m6A modification of *Tbx21* mRNA enhances T-bet expression, promoting CD8^+^ T cell differentiation and effector cytokine production. FTO demethylation of *Fas* mRNA limits apoptosis, while IGF2BP3 stabilizes m6A-modified *Fas* mRNA to promote it.** (B)** Regulation of *TNF*/*CD40L* transcripts in CD4^+^ T cells depends on the balance between FTO and YTHDF2. METTL3-mediated m^6^A modification stabilizes these mRNAs, supporting CD4^+^ T cell activation and cytokine secretion.** (C)** m^6^A modifications orchestrate CD4^+^ T cell lineage fate. METTL3 stabilizes *Tcf-7* mRNA (encoding TCF-1) to drive Tfh cell differentiation and B cell help, whereas METTL14 stabilizes *Foxp3* mRNA to promote iTreg development. Loss of METTL14 disrupts mTOR signaling, favoring Th17 differentiation.** (D)** ALKBH5 demethylation of *Jagged1*/*Notch2* mRNA prevents transcript degradation, sustaining Notch signaling. This promotes pre-γδ T cell proliferation and the differentiation of mature γδ T cells. **(E)** METTL14-mediated m^6^A modification of *Cish* mRNA preserves thymocyte viability by limiting p53/ROS-driven apoptosis. Invariant NKT cells expand upon IL-15 stimulation and α-GalCer injection, producing cytokines including IFN-γ and IL-4.

In the iNKT lineage, METTL14-dependent m^6^A modification is indispensable for developmental programming and survival. Deletion of METTL14 increases apoptosis in double-positive thymocytes, impairs Vα14-Jα18 rearrangement, and markedly reduces iNKT cell numbers. Residual iNKT cells exhibit defective maturation, enhanced apoptosis, and impaired cytokine production due to compromised IL-2/IL-15 responsiveness, diminished TCR signalling, and elevated Cish expression 92. Similarly, METTL3-dependent methylation intrinsically maintains iNKT cell homeostasis and effector specification by stabilizing *Creb1* mRNA. Conditional ablation of METTL3 in CD4^+^CD8^+^ double-positive thymocytes disrupts m^6^A-dependent CREB1 translation and phosphorylation, impairing iNKT cell proliferation, lineage differentiation, and cytokine production, ultimately compromising antitumour immunity against melanoma. Restoration of Creb1 expression rescues these defects, defining a critical m^6^A-CREB1 regulatory axis that orchestrates transcriptional networks controlling iNKT cell development and effector programming 93 **(Figure 1)**. Together, these findings position m^6^A as a key post-transcriptional regulator that integrates thymic developmental signalling, transcription factor networks, and cytokine responsiveness to shape unconventional T cell lineages. Through coordinated actions of its writer and eraser enzymes, m^6^A establishes transcriptional thresholds governing γδ T cell subset composition and iNKT cell maturation, thereby aligning innate-like T cell plasticity with tissue immunity and antitumour defence.

### RNA modifications beyond m^6^A in T cell immunity

Beyond m^6^A, a diverse repertoire of RNA modifications fine-tunes T cell activation, effector programming, and immune tolerance through distinct molecular pathways. The cytosine-5 methyltransferase NSUN2 enhances IL-17A expression by methylating cytosine C466 within *Il17a* mRNA, promoting translational efficiency without altering transcript abundance. Deletion of Nsun2 abolishes homocysteine-induced IL-17A upregulation, establishing a mechanistic link between metabolic stress and T cell-driven inflammation 94.

At the level of Ψ modification, comparative Ψ-seq profiling reveals that Ψ landscapes in primary and immortalized human T cells are highly conserved, with 87% of Ψ sites shared and enriched in transcripts encoding core cellular machinery, including RNA-processing enzymes. Divergent Ψ marks correspond primarily to transcript availability rather than enzymatic expression: Jurkat-specific Ψ sites associate with immune activation and oncogenic pathways, whereas primary T cells display Ψ-modified transcripts related to calcium signalling and vesicular trafficking. Despite similar expression of Ψ synthases, the differential Ψ distribution reflects trans-acting regulatory control that dynamically shapes T cell states across physiological and transformed contexts 95. In innate-immune crosstalk, the Ψ synthase PUS10 modulates tRNA pseudouridylation and fragmentation, thereby maintaining RNA homeostasis. PUS10 deficiency increases tRNA-derived small RNAs and retroelement transcription, generating RNA-DNA hybrids that activate the cGAS-STING pathway. This intrinsic inflammatory loop heightens interferon responses and perturbs immune regulation, altering T cell activation thresholds and promoting autoimmune susceptibility. Supplementation with defined tRNA-derived fragments restores immune balance, identifying PUS10 as a pivotal regulator of RNA-driven inflammatory tone within T cell-relevant networks 96. Chemical modifications of uridine residues can also be synthetically leveraged to modulate T cell activity. Unmodified *in*-*vitro*-transcribed mRNA triggers robust chemokine secretion and lymphocyte recruitment, whereas uridine modifications—such as Ψ, m^1^Ψ, and 5moU—progressively attenuate this immunogenicity. These synthetic nucleoside chemistries allow precise tuning of mRNA-induced immune activation, enhancing the immunosuppressive potential of stromal cells and augmenting *IL-10* mRNA anti-inflammatory efficacy, thereby influencing T cell proliferation and functional polarization 97.

tRNA and mRNA cap modifications further integrate transcriptional activation with translational output during early T cell responses. Antigenic stimulation induces TRMT61A and TRMT6, which catalyse m^1^A^58^ modification on early-expressed tRNAs. These modified tRNAs boost translation of proteins such as MYC, supporting the metabolic and biosynthetic reprogramming required for clonal expansion. Loss of Trmt61a in CD4^+^ T cells reduces MYC synthesis, induces cell-cycle arrest, and limits T cell-mediated pathology in colitis, revealing a tRNA-modification-driven translational program essential for effector activation 10. In parallel, the RNA-cap methyltransferase RNMT promotes ribosome biogenesis via m^7^G capping of transcripts encoding TOP mRNAs and snoRNAs. RNMT upregulation ensures coordinated rRNA and mRNA production for ribosomal assembly, whereas RNMT loss disrupts LARP1-dependent expression, impairs ribosome synthesis, and restricts CD4^+^ T cell proliferation and effector differentiation 98.

**Figure 2:**
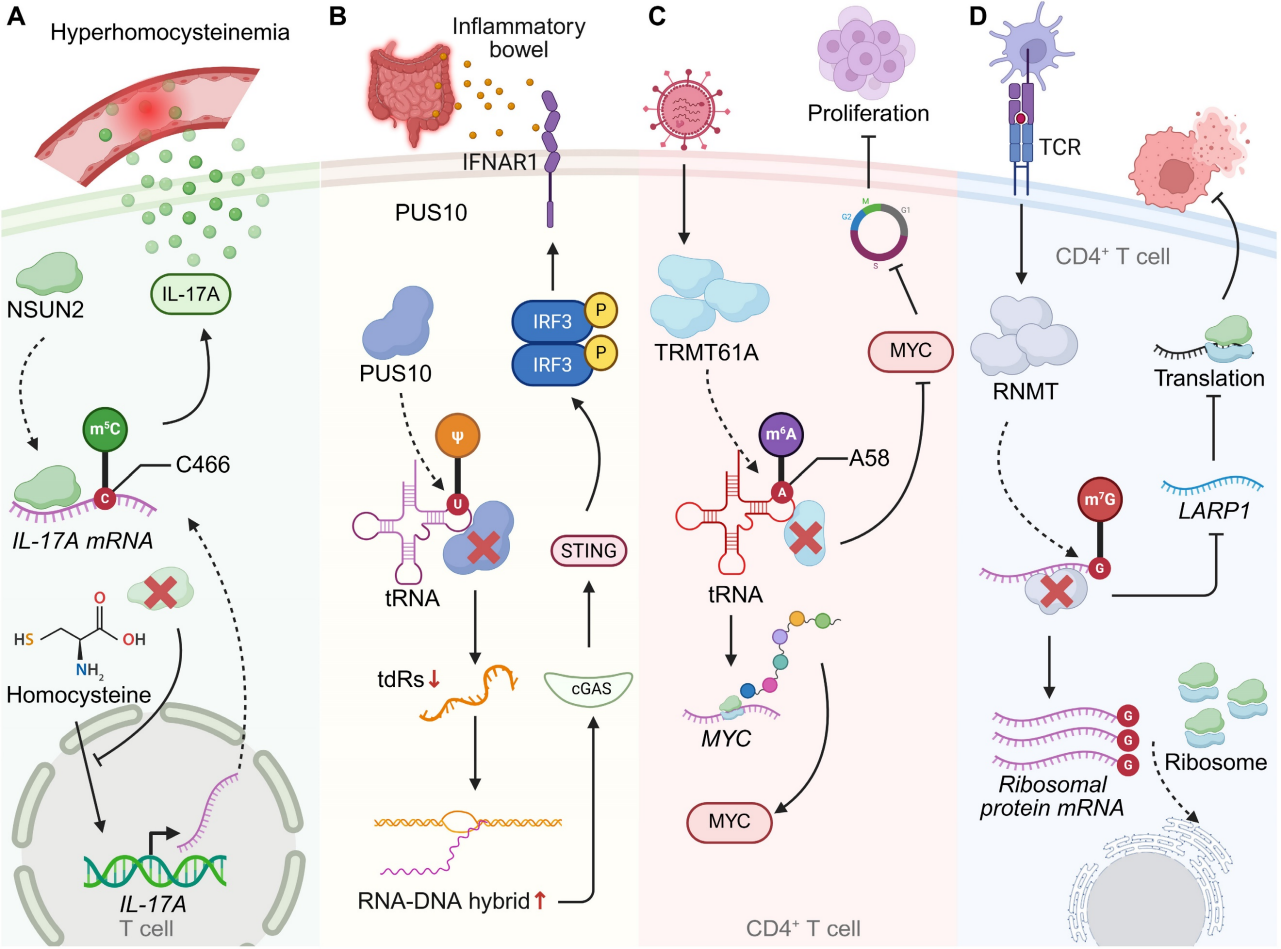
** RNA modifications shape T cell function and immune regulation under pathological conditions. (A)** In hyperhomocysteinemia, NSUN2 catalyzes m^5^C modification of* IL-17A* mRNA at cytosine 466, enhancing its translational efficiency and promoting IL-17A production in T cells. NSUN2 deficiency blocks the homocysteine-driven increase in IL-17A, highlighting a link between metabolic stress and T cell-mediated inflammatory responses. **(B)** PUS10-mediated pseudouridylation of tRNA regulates innate immune sensing. Loss of PUS10 reduces tRNA stability, increases RNA-DNA hybrid formation, and activates the cGAS-STING pathway, leading to inflammatory signaling. This mechanism contributes to intestinal immune dysregulation in inflammatory bowel disease (IBD). **(C)** TRMT61A installs m^1^A at position A_58_ of tRNA, thereby sustaining MYC expression in CD4^+^ T cells. MYC-driven transcriptional programs promote cell proliferation and clonal expansion, coupling RNA modification to T cell metabolic and proliferative fitness. **(D)** RNMT-dependent m^7^G modification of ribosomal protein mRNAs regulates CD4^+^ T cell activation. In response to TCR stimulation, RNMT and LARP1 coordinate ribosome biogenesis and translation, thereby enabling efficient protein synthesis during T cell responses.

Collectively, these findings delineate a multilayered epitranscriptomic network in which diverse RNA modifications cooperate to orchestrate T cell development, activation, and functional plasticity. Acting at multiple hierarchical levels—from tRNA charging and mRNA translation to cytokine secretion and innate-immune feedback—these chemical marks integrate metabolic cues, stress signals, and immune pathways into coherent post-transcriptional programs. This integrated framework endows T cells with the capacity to rapidly reconfigure gene expression in response to environmental and immunological perturbations, sustaining immune homeostasis while preventing uncontrolled activation or tolerance breakdown 99 **(Figure 2, Table 1)**.

### Potential epitranscriptomic crosstalk integrates RNA modification networks in T cell immunity

Epitranscriptomic modifications have emerged as critical regulators of immune homeostasis, adding a dynamic layer of control that complements transcriptional and translational programs. Among the diverse RNA modifications identified to date, examples such as m^6^A and m^5^C illustrate how chemical marks on RNA can fine-tune transcript fate and coordinate cellular adaptation to stress. Once viewed as discrete regulatory systems, these modification pathways are now recognized to engage in extensive cross-regulatory interactions. Reciprocal modification of their effector transcripts forms intricates post-transcriptional feedback loops, while coordinated expression of regulators such as the m^6^A eraser ALKBH5 and the m^5^C writer NSUN4 links RNA methylation to proteasomal turnover, mitochondrial function, and post-translational signaling networks 100. This interplay reveals a hierarchical control system in which distinct RNA marks converge to modulate gene expression in a context-dependent manner.

Within the immune system, such crosstalk provides a conceptual basis for understanding how metabolic and environmental cues are integrated at the RNA level to calibrate T cell activation, differentiation, and effector function. The coordination between different RNA modification machineries may enable T cells to dynamically balance biosynthetic demands, redox homeostasis, and cytokine production during immune activation. Supporting this notion, the cooperative m^6^A and m^5^C modification of GPX4 by RBM15B, IGFBP2, and NSUN5 sustains redox equilibrium and activates the cGAS-STING pathway, thereby enhancing antitumor immune responses 101. By analogy, similar dual-modification networks may operate in T cells to link mitochondrial metabolism and RNA stability with immune signaling precision. These insights collectively point to potential RNA modification crosstalk as an emerging axis of immune regulation—one that integrates metabolic resilience with epitranscriptomic plasticity to fine-tune T cell-mediated immunity and therapeutic responsiveness.

## RNA modification-driven remodeling of T cell immunity across disease contexts

### RNA modification-mediated regulation of CD8^+^ T cell function in cancer

**Figure 3:**
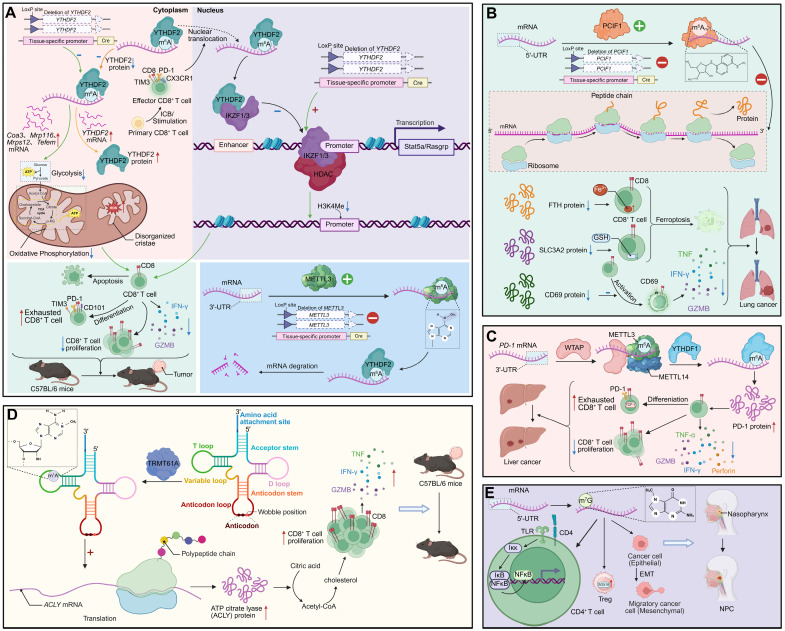
** RNA modifications regulate CD8^+^ T cell-mediated antitumor immunity and shape the immunosuppressive tumor microenvironment. (A)** YTHDF2 controls mitochondrial metabolism and epigenetic programming of CD8^+^ T cells. Conditional deletion of Ythdf2 impairs oxidative phosphorylation, disrupts cristae structure, and upregulates genes such as *Cox3*, *Mtp16*, and *Tefm*. Nuclear YTHDF2 stabilizes IKZF1/3 transcriptional activity and represses HDAC-mediated H3K4me modifications, thereby sustaining effector function. Loss of YTHDF2 accelerates CD8^+^ T cell exhaustion, with increased PD-1, TIM3, and CD101 expression, impaired proliferation and tumor progression. METTL3-dependent m^6^A modification further stabilizes transcripts critical for effector CD8^+^ T cell activity, whereas YTHDF2 deletion promotes mRNA decay. **(B)** PCIF1 regulates m^6^A_m_ modification and translation in CD8^+^ T cells. PCIF1-mediated m^6^A_m_ modification diminishes ribosome-associated peptide synthesis, lowering proteins such as FTH, SLC3A2 and CD69. This triggers ferroptosis and compromises CD8^+^ T cell cytotoxicity, reducing TNF, IFN-γ, and GZMB production, and accelerating lung cancer progression. **(C)** METTL3-METTL14-YTHDF1 complex maintains effector function in liver cancer. m^6^A modification of *Pdcd1* (PD-1) transcripts enhances PD-1 protein expression, promoting CD8^+^ T cell exhaustion. YTHDF1 stabilizes effector transcripts, sustaining cytotoxicity (TNF, IFN-γ, perforin, GZMB), while WTAP regulates m^6^A deposition. Dysregulation of this axis reduces CD8^+^ T cell proliferation and accelerates immune dysfunction. **(D)** TRMT61A installs m^1^A modification on tRNA, regulating translation of ACLY and metabolic reprogramming in CD8^+^ T cells. m^1^A at wobble positions enhances ACLY-driven acetyl-CoA production, fueling cholesterol and citric acid metabolism. This sustains effector function and cytokine release (TNF, IFN-γ, GZMB), enabling tumor control in C57BL/6 mice. **(E)** m^7^G modification shapes regulatory T cell (Treg) stability and epithelial-mesenchymal transition (EMT) in the tumor microenvironment of NPC. m^7^G-modified transcripts activate the IKK-NFκB pathway in CD4^+^ T cells, promoting Treg differentiation. In parallel, RNA modification stabilizes EMT-associated transcripts, driving cancer cell migration and NPC progression.

In the tumor microenvironment, RNA modifications act as critical determinants of CD8^+^ T cell persistence, metabolic fitness, and responsiveness to immunotherapy, with distinct regulatory enzymes exerting either supportive or suppressive effects on antitumor immunity 102-104. YTHDF2 exemplifies a positive regulator by sustaining polyfunctionality in early effector-like CD8^+^ T cells through facilitation of nascent RNA synthesis and modulation of chromatin architecture. High YTHDF2 expression is indispensable for preserving mitochondrial fitness and maintaining cytotoxic potential, whereas its loss accelerates tumor progression, compromises effector responses, and diminishes the efficacy of PD-1 blockade. Mechanistically, YTHDF2 interacts with IKZF1/3 to maintain transcription of key target genes, and pharmacological intervention with lenalidomide can rescue these defects, underscoring the therapeutic relevance of targeting RNA modification pathways 18. In contrast, the m^6^A_m_ methyltransferase PCIF1 functions as a negative regulator of CD8^+^ T cell antitumor activity. Deletion of Pcif1, either systemically or specifically in T cells, not only reduces tumor burden but also increases the infiltration of activated cytotoxic CD8^+^ T cells. This effect is mediated by the upregulation of m^6^A_m_ -modified ferroptosis suppressor genes such as *Fth1* and *Slc3a2*, along with the activation marker Cd69, thereby enhancing resistance to ferroptosis and augmenting antitumor function. Pcif1 deficiency also potentiates the efficacy of anti-PD-1 therapy and improves tumor control in CAR-T cell models, with clinical data linking low PCIF1 expression to superior immunotherapy outcomes 105. Complementing these findings, the m^6^A methyltransferase adaptor WTAP contributes to the onset of CD8^+^ T cell exhaustion in hepatocellular carcinoma (HCC) by increasing *PD-1* mRNA translation in a YTHDF1-dependent manner. Elevated WTAP expression in tumor-infiltrating CD8^+^ T cells enhances PD1 levels, suppressing proliferation and effector activity, whereas WTAP silencing—especially in combination with PD1 knockdown—restores immune function, inhibits tumor growth, and improves responses to PD-1 blockade. Together, these examples illustrate how distinct m^6^A and m^6^A_m_ regulators orchestrate the balance between functional resilience and exhaustion in CD8^+^ T cells, ultimately shaping antitumor immunity and therapeutic responsiveness. Notably, the tRNA m^1^A methyltransferase TRMT61A enhances CD8^+^ T cell antitumor immunity by promoting cholesterol biosynthesis. TRMT61A-driven m^1^A modification facilitates translation of ATP citrate lyase, a key enzyme in the cholesterol synthesis pathway, supporting T cell proliferation and cytotoxic function. Loss of TRMT61A impairs cholesterol production, leading to diminished effector responses and tumor control, which can be restored by cholesterol supplementation 106. These findings position tRNA m^1^A as a crucial metabolic regulator linking translational control to the functional competence of CD8^+^ T cells in the tumor microenvironment. Single-cell transcriptomic profiling of nasopharyngeal carcinoma (NPC) reveals that m^7^G RNA modification is intricately linked to immune dysregulation within the tumor microenvironment. CD4^+^ and CD8^+^ T cells in NPC exhibit reduced m^7^G scores compared to nonmalignant tissues, alongside altered expression of immune co-stimulatory and co-inhibitory molecules, suggesting impaired antitumor function. m^7^G-associated interactions between T cells and other stromal and immune populations, including macrophages and fibroblasts, further reshape the immunosuppressive milieu 107 **(Figure 3)**.

### RNA modification-mediated regulation of CD4^+^ T cell function in diseases

#### Autoimmune diseases

##### SLE

In SLE, CD4^+^ T cells undergo extensive remodeling of RNA modifications, encompassing m^6^A, m^5^C, and ac^4^C marks on both nuclear- and mitochondrial-encoded transcripts. These modifications collectively integrate transcriptional, metabolic, and post-transcriptional networks that shape lineage commitment, effector activity, and pathogenic potential 108.

METTL3 functions as a pivotal regulator of CD4^+^ T cell activation and lineage commitment in SLE. Reduced METTL3 expression correlates with aberrant activation dynamics and skewed effector differentiation. Pharmacological inhibition of METTL3 destabilizes *Foxp3* mRNA by impairing m^6^A-dependent protection, thereby limiting regulatory T cell development and favoring pro-inflammatory responses. *In vivo*, METTL3 blockade augments CD4^+^ T cell activation, diminishes Treg differentiation, enhances antibody production, and exacerbates lupus-like pathology 109. Mitochondrial dysfunction further links m^6^A dysregulation to pathogenic activation. The mitochondrial transcript MT-ND6 exhibits hypermethylation and reduced expression in SLE CD4^+^ T cells, correlating with disease activity and autoantibody titres. MT-ND6 deficiency impairs ATP production, increases total and mitochondrial ROS, and activates inflammatory transcriptional programs—defects reversible by mitochondrial antioxidant treatment—demonstrating that m^6^A safeguards mitochondrial integrity to restrain pathogenic activation 110.

Beyond m^6^A, SLE CD4^+^ T cells display widespread perturbation of m^5^C and ac^4^C marks. Global m^5^C levels are reduced despite an increased number of m^5^C-modified transcripts, particularly near translation initiation sites, implicating translational dysregulation in aberrant immune activation. Downregulation of NSUN2 contributes to this disrupted methylation pattern and altered mRNA metabolism 111. In parallel, reduced ac^4^C and its writer NAT10 coincide with altered acetylation of coding sequences enriched in metabolic and NF-κB signalling genes. Dysregulated ac^4^C distribution on immune-regulatory transcripts such as *USP18*, *GPX1*, and *RGL1* affects mRNA stability and translation, linking ac^4^C remodeling to oxidative stress and hyperinflammatory states 112. Together, these findings define a multilayered epitranscriptomic landscape in which m^6^A, m^5^C, and ac^4^C cooperatively modulate transcriptional fidelity, mitochondrial function, and redox homeostasis to shape autoimmune pathology in SLE.

##### Psoriasis

Psoriasis is a chronic inflammatory skin disorder driven by Th17-mediated pathology. In CD4^+^ T cells, enhanced m^6^A deposition on *Il17a* transcripts increases their stability and abundance, amplifying IL-17A production and psoriatic inflammation. Loss of the demethylase ALKBH5 exacerbates disease severity, whereas METTL3 ablation alleviates pathology, underscoring that m^6^A-dependent stabilization of *Il17a* mRNA drives Th17 expansion and disease progression 113.

##### Ocular immune-mediated disorders

In ocular autoimmunity, including Graves' ophthalmopathy (GO) and autoimmune uveitis, aberrant m^6^A modification reinforces dysregulated CD4^+^ T cell metabolism and inflammatory programming. In GO, WTAP expression is markedly upregulated in CD4^+^ T cells, promoting m^6^A deposition on *THBS1* transcripts. This hypermethylation stabilizes THBS1, driving glycolytic flux—reflected by increased glucose uptake and lactate production—thereby enhancing Th17 differentiation and suppressing Treg induction. Silencing WTAP reduces THBS1 methylation, normalizes metabolism, and restores T cell balance, defining a WTAP-THBS1-glycolysis axis as a central driver of GO pathology 114. Conversely, in experimental autoimmune uveitis, METTL3 plays a protective role. Reduced METTL3 expression and global m^6^A levels correlate with heightened inflammation, whereas enforced METTL3 expression mitigates disease. Mechanistically, METTL3 promotes YTHDC2-dependent m^6^A modification of *ASH1L* transcripts, stabilizing them and suppressing IL17 and IL23R expression, thereby limiting Th17 effector activity and ocular inflammation 115. These dual mechanisms illustrate how m^6^A-dependent reprogramming can either amplify or restrain CD4^+^ T cell pathogenicity, depending on the molecular context.

##### IBD (ulcerative colitis)

**Figure 4:**
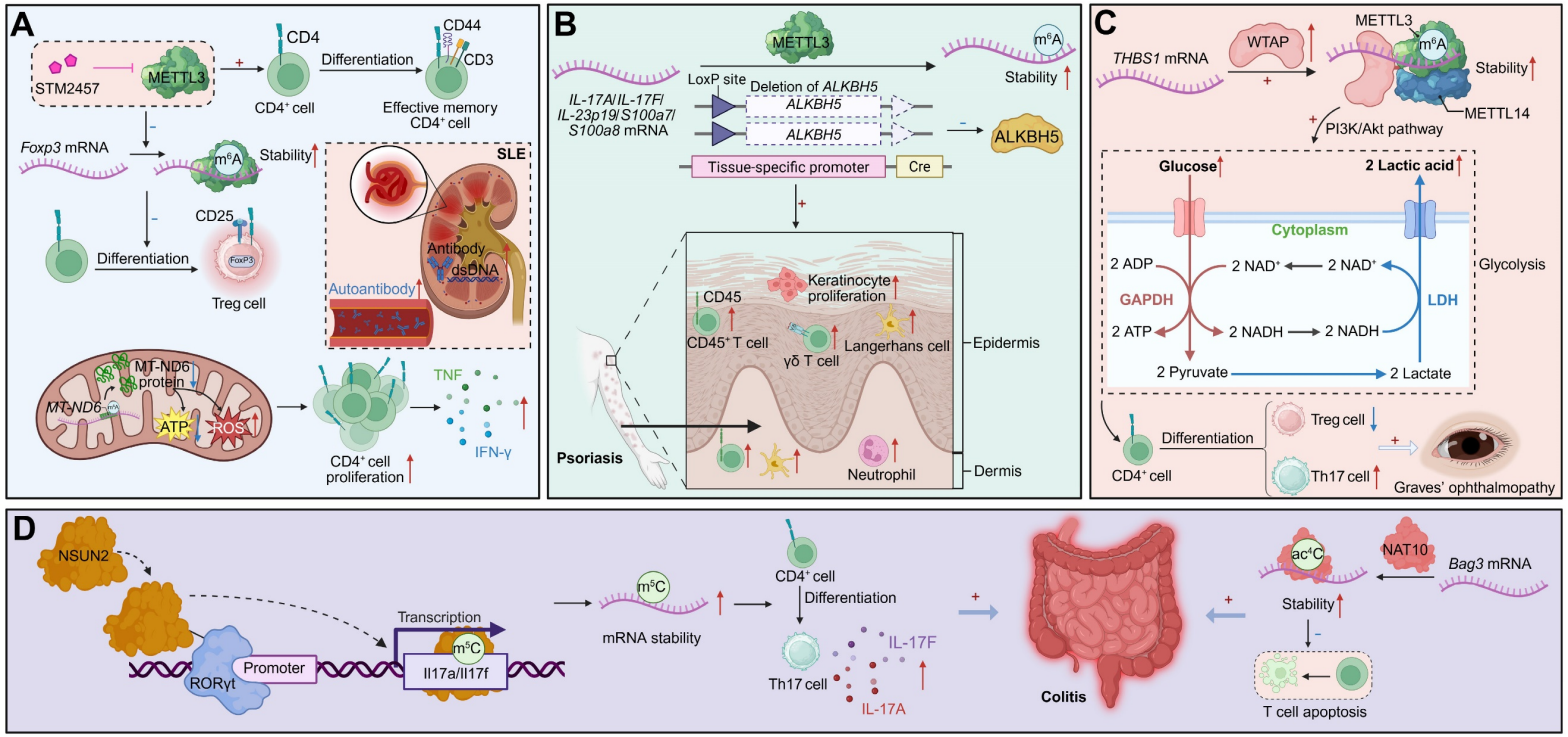
** RNA modifications regulate CD4^+^ T cell differentiation and autoimmune pathogenesis. (A)** METTL3-mediated m^6^A modification of *Foxp3* mRNA promotes its stability and supports Treg differentiation, a process that can be pharmacologically inhibited by STM2457. Concurrently, METTL3-mediated m^6^A modification of *MT-ND6* mRNA reduces its expression, leading to mitochondrial dysfunction (ATP loss, ROS accumulation), aberrant CD4^+^ T cell proliferation, and cytokine production. In SLE, impaired Treg function and mitochondrial defects contribute to enhanced autoantibody production, and systemic inflammation. **(B)** Deletion of ALKBH5 enhances METTL3-mediated m^6^A methylation on pro-inflammatory transcripts (*IL-17A*, *IL-17F*, *IL-23p19*, *S100a7/8*), increasing their stability. This drives keratinocyte hyperproliferation, epidermal thickening, and infiltration of inflammatory cells (CD45^+^ T cells, γδ T cells, neutrophils, Langerhans cells) in psoriasis. **(C)** WTAP-METTL3-METTL14 complex stabilizes *THBS1* mRNA via m^6^A modification, activating the PI3K-Akt pathway and reinforcing glycolysis. This metabolic rewiring skews CD4^+^ T cell differentiation toward Th17 cells at the expense of Tregs, exacerbating disease manifestations such as GO. **(D)** NSUN2-mediated m^5^C modification of *Il17a*/*Il17f* transcripts enhances their stability, driving Th17 differentiation and excessive IL-17A/F secretion, which exacerbates colitis. Additionally, NAT10-catalyzed ac^4^C modification stabilizes *Bag3* mRNA, preventing T cell apoptosis and sustaining inflammation.

In ulcerative colitis, sustained T cell-driven inflammation is maintained by cooperative actions of ac^4^C and m^5^C modifications. The acetyltransferase NAT10 catalyzes ac^4^C modification on *Bag3* mRNA, stabilizing this anti-apoptotic transcript and supporting pathogenic CD4^+^ T cell survival. NAT10 deletion accelerates apoptosis and diminishes T cell persistence, thereby reducing colitogenic potential 116. Concurrently, the methyltransferase NSUN2 promotes Th17 differentiation by forming a complex with RORγt to methylate *Il17a* and *Il17f* transcripts, enhancing their stability and cytokine production. NSUN2 deficiency impairs Th17 polarization and ameliorates colitis, revealing a RORγt-NSUN2 axis that sustains Th17 effector function 117. Collectively, ac^4^C- and m^5^C-mediated programs integrate metabolic, survival, and cytokine circuits to maintain chronic inflammation in ulcerative colitis, exemplifying how diverse RNA modifications converge to stabilize pro-inflammatory states** (Figure 4)**.

#### Transplantation and graft rejection

In kidney transplantation, epitranscriptomic remodeling of CD4^+^ T cells critically determines the equilibrium between immune tolerance and graft rejection. In states of operational tolerance, the m^6^A methyltransferase component WTAP is upregulated in CD4^+^ T cells and correlates with expansion of the Treg compartment. Mechanistically, WTAP promotes m^6^A deposition within the coding region of *Foxo1* mRNA, stabilizing its transcript and sustaining FOXO1 expression. This enhances Treg differentiation and suppressive activity, thereby maintaining peripheral tolerance. *In vivo*, WTAP overexpression prolongs allograft survival by augmenting Treg-mediated immunoregulation and dampening inflammatory activation, establishing a WTAP-Foxo1-m^6^A axis as a central safeguard of transplant tolerance 118. Conversely, during allograft rejection, alloreactive CD4^+^ T cells exhibit globally elevated m^6^A modification, partly driven by METTL3. Pharmacological inhibition of METTL3 using STM2457 reduces overall m^6^A levels, inducing G₀ cell-cycle arrest, increasing apoptosis, and impairing both proliferation and effector differentiation of polyclonal and alloantigen-specific CD4^+^ T cells. These effects are accompanied by attenuated Th1 polarization and downregulation of key transcriptional drivers, including Ki-67, c-Myc, and T-bet 119. Collectively, these findings delineate a dual role for m^6^A-dependent regulation in transplantation: promoting tolerance through Treg stabilization while sustaining rejection by supporting effector T cell fitness and persistence. This functional dichotomy positions the RNA methylation machinery as a tunable checkpoint in graft-specific immunity—one that could be therapeutically manipulated to tip the balance between durable tolerance and immune rejection.

#### Infectious diseases

Epitranscriptomic regulation has emerged as a pivotal determinant of CD4^+^ T cell fate and function during infection 120, 121. In acute bacterial challenge, the m^6^A demethylase FTO safeguards the Th1 differentiation program by maintaining Tbx21 (T-bet) expression and sustaining IFN-γ production. Genetic ablation of FTO leads to defective antigen-specific Th1 expansion, resulting in impaired pathogen clearance in both *in vitro* and *in vivo* models. Mechanistically, FTO dynamically modulates the stability and translational efficiency of lineage-defining transcripts, thereby coordinating transcriptional and metabolic programs that underpin effective antibacterial immunity 122. A comparable dependence on m^6^A regulation is observed in chronic viral infection, where reactivation of latent HIV-1 in CD4^+^ T cells induces a transient but pronounced increase in global m^6^A RNA methylation, independent of changes in writer or eraser abundance. Silencing m^6^A methyltransferases or pharmacologically inhibiting methylation markedly suppresses HIV-1 reactivation, demonstrating that m^6^A facilitates viral transcriptional reawakening. m^6^A-seq profiling reveals widespread remodeling of both host and viral transcript methylation during latency reversal, suggesting that RNA methylation reprograms the T cell transcriptome to favor viral gene expression and reservoir destabilization 123. In acute viral infections, particularly COVID-19, altered m^6^A landscapes in peripheral blood leukocytes correlate with CD4^+^ T cell activation states and clinical outcomes. Unsupervised clustering of patient transcriptomes reveals two distinct m^6^A-modification patterns: an activation-enriched cluster characterized by enhanced T cell effector signatures and improved recovery, and a metabolically reprogrammed, checkpoint-suppressed cluster marked by reduced m^6^A scores and poor prognosis. A nine-gene m^6^A-related transcriptional signature derived from these datasets reliably predicts disease trajectory, linking the intensity and quality of antiviral responses to the m^6^A regulatory landscape 124. Collectively, these studies identify m^6^A-dependent post-transcriptional regulation as a unifying mechanism that enables CD4^+^ T cells to tailor their effector programs across diverse infectious contexts. By coupling transcriptional activation with translational control, m^6^A modification integrates metabolic readiness, cytokine production, and viral sensing into a coherent immune response, thereby shaping both pathogen clearance and the persistence of infection.

#### Neuroinflammatory and CNS autoimmune disorders

**Figure 5:**
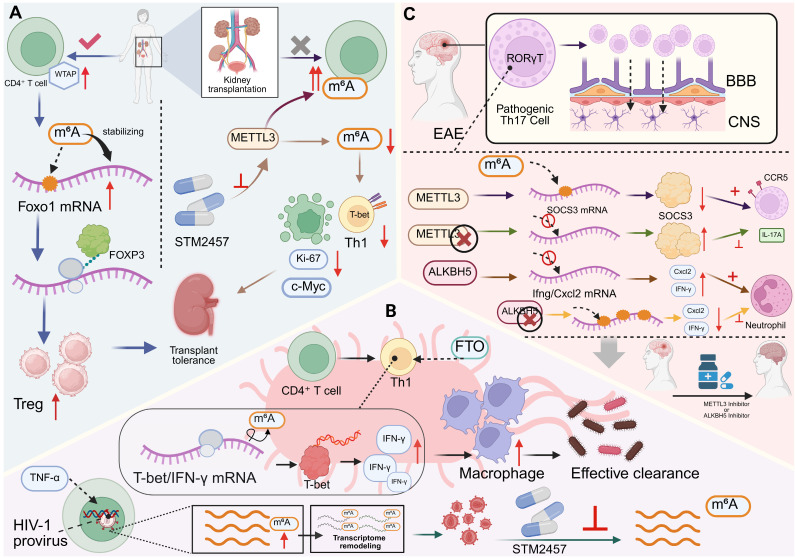
** m^6^A RNA modifications orchestrate CD4^+^ T cell fate and immune-mediated disease. (A)** The dual role of m^6^A RNA modification in transplantation. The WTAP-m^6^A-FOXO1 axis promotes Treg-mediated tolerance and graft survival. In contrast, METTL3-mediated m^6^A modification enhances effector T cell proliferation, differentiation, and function, driving rejection. Targeting METTL3 can inhibit its activity through drugs such as STM2457, reduce T-bet, Ki-67, and c-Myc to suppress Th1 polarization, while enhancing Treg responses, thereby inhibiting this alloreactive response. **(B)** RNA modification fine-tunes CD4^+^ T cell responses to infections. (Top) FTO-mediated demethylation regulates Th1-driven macrophage activation. By controlling m^6^A on *T-bet*/*IFN-γ* transcripts, FTO enhances IFN-γ secretion, which augments macrophage effector functions and pathogen clearance. (Bottom) In HIV-1 latency, reactivation signals induce m^6^A methylation on viral and host transcripts, promoting viral gene expression and reactivation, which can be blocked by METTL3 inhibition. **(C)** m^6^A modifications influence Th17 pathogenicity in autoimmune disease. In EAE, the presence of METTL3 reduces the stability of *SOCS3* mRNA, which in turn leads to increased expression of IL-17A and CCR5, the m^6^A demethylase ALKBH5 preserves the stability of pro-inflammatory transcripts such as *Ifng* and *Cxcl2*, thereby maintaining CD4^+^ T cell effector potential and promoting neutrophil recruitment during neuroinflammation. Pharmacological inhibition of METTL3 or ALKBH5 impairs Th1 responses and reduces immune clearance, underscoring therapeutic potential of m^6^A modulators.

Neuroinflammatory and central nervous system (CNS) autoimmune diseases—exemplified by experimental autoimmune encephalomyelitis (EAE)—are critically governed by m^6^A-dependent regulation of pathogenic CD4^+^ T cell subsets. m^6^A RNA methylation reinforces Th17 lineage integrity and effector persistence in CNS autoimmunity: deletion of the methyltransferase METTL3 in total T cells or specifically in Th17 cells markedly reduces CNS infiltration and ameliorates disease severity. Mechanistically, loss of METTL3 disrupts m^6^A-dependent decay of *Socs3* mRNA, leading to its stabilization and the suppression of Il17a and Ccr5 expression, thereby dismantling the Th17 transcriptional circuit essential for pathogenic activity 125. Conversely, the m^6^A demethylase ALKBH5 sustains inflammatory effector programs by preventing methylation-dependent degradation of proinflammatory transcripts such as *Ifng* and *Cxcl2*. ALKBH5 ensures their stability, maintaining cytokine output and promoting neutrophil recruitment within inflamed CNS tissues. In its absence, hypermethylation of these transcripts accelerates decay, dampens effector cytokine production, and mitigates autoimmune pathology 126. Together, these findings delineate a bidirectional m^6^A-regulatory circuit that dynamically balances methylation-driven transcript turnover and demethylation-mediated transcript stabilization. This interplay fine-tunes cytokine networks and effector persistence, preserving the identity and inflammatory tone of CNS-infiltrating CD4^+^ T cells. Such dynamic epitranscriptomic regulation integrates transcriptional feedback, metabolic adaptation, and immune signaling to orchestrate neuroinflammatory responses, highlighting RNA methylation machinery as a potential therapeutic target in CNS autoimmune disease **(Figure 5)**.

#### Other diseases

Beyond classical autoimmune, infectious, and neuroinflammatory disorders, RNA modifications in CD4^+^ T cells contribute to the pathogenesis of diverse immune-related conditions. In asthma, skewed differentiation toward Th2 and Th17 lineages is coupled to metabolic reprogramming through m^6^A-dependent control. METTL3-mediated m^6^A methylation inhibits processing of pri-miR-192-5p, thereby relieving repression of stearoyl-CoA desaturase 1 (SCD1). Elevated SCD1 promotes lipid metabolic adaptation that increases Th2 and Th17 frequencies while limiting Th1 cells. Restoration of miR-192-5p or pharmacological inhibition of SCD1 reverses this lineage bias and mitigates airway inflammation, whereas oleic-acid supplementation intensifies the inflammatory phenotype 127. In parallel, METTL3-driven m^6^A deposition within the 3′ UTR of *Foxp3* mRNA enables YTHDF2-mediated degradation, reducing Treg proportions and shifting cytokine secretion toward a pro-inflammatory profile 128. Aberrant m^6^A methylation in CD4^+^ T cells also forms a shared epitranscriptomic signature linking coronary artery disease (CAD) and invasive ductal carcinoma (IDC). Comparative profiling identifies dysregulated m^6^A marks on inflammation-associated transcripts, including *VEGFA* and *AIMP1*, in both conditions. CRISPR-Cas9-mediated modulation of BRCA1 alters m^6^A distribution, influences R-loop formation and DNA-damage susceptibility, and modulates p53 activity, thereby affecting immune-tumour interactions and enhancing cytotoxic T lymphocyte-mediated killing of breast-cancer cells 129. Together, these findings underscore that RNA modification-dependent control of T cell fate and function extends beyond canonical autoimmune and infectious diseases, bridging metabolic, inflammatory, and oncogenic pathways across distinct pathological contexts.

### RNA modification-mediated regulation of unconventional T cell function in diseases

**Figure 6:**
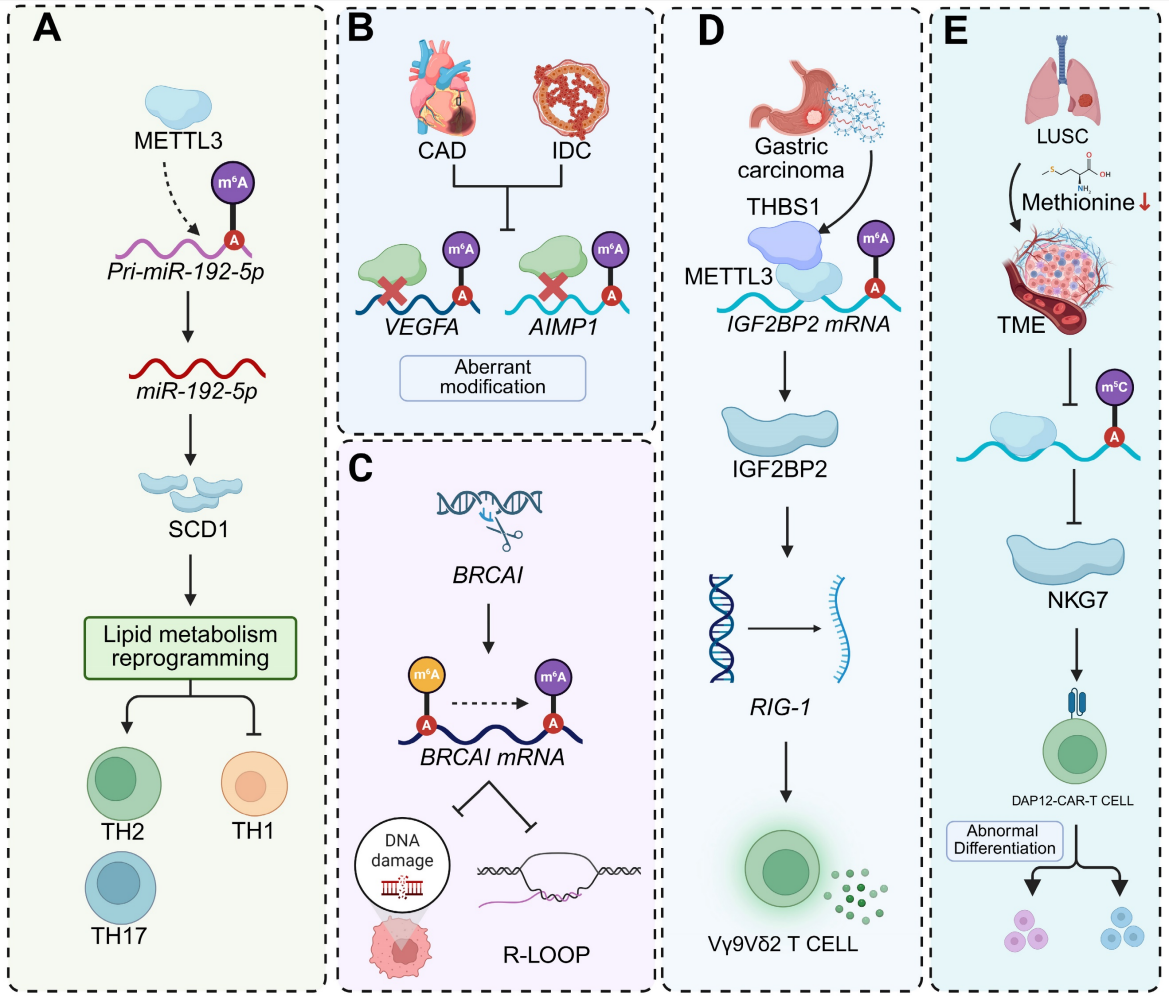
** Schematic representations of RNA modification (m^6^A, m^5^C)-mediated regulatory mechanisms in diverse pathological contexts. (A)** METTL3-catalyzed m^6^A modification of pri-miR-192-5p promotes its processing into mature miR-192-5p. This miR-192-5p then targets SCD1, triggering lipid metabolism reprogramming and shaping the functional phenotypes of Th1, Th2, and Th17 cells. **(B)** In CAD and IDC, abnormal m^6^A modifications occur on the mRNAs of VEGFA and AIMP1, implying dysregulated post-transcriptional regulation in these cardiovascular disorders. **(C)** During BRCA1 transcription, m^6^A modification on *BRCA1* mRNA modulates R-loop formation, thereby influencing the cellular response to DNA damage, a process crucial for maintaining genomic stability. **(D)** In gastric carcinoma, THBS1-associated METTL3 mediates m^6^A modification of *IGF2BP2* mRNA, enhancing IGF2BP2 expression. Subsequently, IGF2BP2 regulates RIG-1, which in turn impacts the functional activity of Vγ9Vδ2 T cells in anti-tumor immunity. **(E)** In LUSC, reduced methionine levels affect m^5^C modifications. This alteration regulates NKG7 expression and leads to the abnormal differentiation of DAP12-CAR-T cells within the tumor microenvironment (TME).

Unconventional T cell subsets, such as Vγ9Vδ2 T cells and CAR-T cells, are emerging as crucial mediators of antitumor immunity, with their activation and persistence tightly governed by RNA modifications and metabolic cues. In gastric cancer, tumor-derived exosomal THBS1 enhances the cytotoxicity and effector cytokine production of Vγ9Vδ2 T cells by remodeling the m^6^A RNA methylation landscape. Mechanistically, THBS1 directly interacts with the methyltransferase METTL3 and induces expression of the m^6^A reader IGF2BP2, leading to stabilization of transcripts encoding RIG-I-like receptor signaling components, including *IRF7*, *ISG15*, and *PKR*. This enhanced transcript stability sustains antiviral signaling and effector activation, whereas pharmacological inhibition of the m^6^A machinery abolishes THBS1-driven functional gains 130. In parallel, B7H3-targeted DAP12-CAR-T cells exhibit strong cytotoxicity against lung squamous cell carcinoma (LUSC) but are metabolically constrained within methionine-depleted tumor microenvironments. Nutrient restriction reduces m^5^C RNA modification and downregulates NKG7, a key cytolytic effector gene, thereby driving CAR-T cells toward functional exhaustion. Restoration of NKG7 expression or blockade of tumor SLC7A5-mediated methionine uptake rescues CAR-T cell cytotoxicity under nutrient stress, linking methionine availability and RNA methylation to effector competency 131. Together, these findings define RNA modification as a dynamic regulatory layer that integrates metabolic inputs and tumor-derived signals to fine-tune the effector programs of unconventional T cells. By coupling post-transcriptional control with metabolic adaptation, m^6^A and m^5^C modifications determine whether γδ T cells and CAR-T cells sustain cytotoxic persistence or enter functional exhaustion, underscoring the therapeutic potential of targeting epitranscriptomic-metabolic crosstalk to enhance T cell-based immunotherapies **(Figure 6, Table 2)**.

## Challenges and perspectives

Despite remarkable advances in uncovering the epitranscriptomic regulation of T cell biology, a mechanistic and clinically actionable understanding remains incomplete. Knowledge gaps persist across multiple layers—from modification dynamics and functional hierarchies to clinical translation—largely because existing data are context-specific, technically fragmented, and lack longitudinal resolution. Bridging these gaps will require the convergence of technological innovation, mechanistic dissection, and translational standardization.

### Defining the spatiotemporal landscape of RNA modifications in T cells

Although transcriptional and epigenetic trajectories of T cell differentiation have been resolved at single-cell resolution, an equivalent atlas for RNA modifications is still lacking. Most current studies examine individual marks within limited subsets, without capturing developmental continuity or microenvironmental influences. This piecemeal approach obscures stage-specific dependencies, as the same modification may promote proliferation during activation but suppress memory maintenance later.

To overcome these limitations, single-cell and direct RNA sequencing technologies should be systematically integrated. Nanopore DRS now enables single-molecule, single-nucleotide identification of multiple RNA modifications, offering a unified framework for quantitative profiling 133. Deep-learning frameworks such as modCnet further improve precision by distinguishing diverse cytidine modifications (e.g., ac^4^C and m^5^C) from a single sample, thus revealing combinatorial modification patterns 66. For immunological applications, clinical standardization of single-cell epitranscriptomics will require three interdependent components: (1) reference cell lines and synthetic spike-in controls to calibrate detection sensitivity and ensure reproducibility across laboratories 134; (2) automated, low-input library preparation workflows that integrate DRS with optimized barcoding and multiplexing for small patient samples 135; and (3) harmonized computational pipelines and annotation standards for basecalling, signal normalization, and modification calling 136. Together, these developments will enable construction of a spatiotemporal “T cell epitranscriptomic atlas,” facilitating causal analysis of stage-specific RNA-modification events through inducible perturbation models.

### Capturing early epitranscriptomic signatures of T cell exhaustion

T cell exhaustion in chronic infection and cancer reflects progressive functional decline accompanied by stable epigenetic remodeling. Current markers—based on inhibitory receptors or transcriptional states—identify exhaustion only after irreversible dysfunction has occurred 137, 138. Whether changes in RNA modifications precede these fixed states, acting as early molecular sentinels of exhaustion, remains unresolved. To clarify this, longitudinal single-cell epitranscriptomic profiling should be employed to trace modification dynamics across the exhaustion continuum *in vivo* 139, 140. Integration of these datasets with transcriptomic, metabolic, and chromatin-accessibility profiles would identify reversible nodes that define pre-exhaustion states 141. Transient perturbation of RNA-modifying enzymes at these early time points—via CRISPR interference or programmable RNA editors—can establish causality and identify modifiable checkpoints 142, 143. Mapping such temporal dynamics will yield predictive biomarkers for immune decline and guide the timing of therapeutic interventions to sustain durable effector competence.

### Translating RNA-modification biology into clinical and therapeutic contexts

The translation of RNA-modification biology into clinical applications is still in its infancy, constrained by technical, mechanistic, and safety challenges. Two major barriers hinder its progress: the lack of standardized, clinically compatible assays for limited immune samples, and the difficulty of safely modulating RNA-modifying enzymes whose pleiotropic functions extend across multiple cell types.

To address the first challenge, low-input, high-sensitivity detection platforms must be adapted for clinical workflows. Nanopore-based DRS enables single-molecule, single-nucleotide identification of multiple RNA modifications without chemical pretreatment, providing an efficient framework for clinical translation 143. Integration of machine-learning-driven basecalling algorithms and harmonized cloud-based analysis pipelines will allow reproducible quantification and regulatory standardization 144. Establishing clinical SOPs for RNA isolation, library preparation, and data interpretation will further facilitate the incorporation of RNA-modification biomarkers into patient monitoring and immune stratification programs.

Therapeutically, pharmacologic targeting of RNA-modifying enzymes is emerging as a promising approach for cancer immunotherapy. The oral METTL3 inhibitor STC-15 exemplifies this strategy: METTL3 inhibition enhances interferon signalling and dsRNA accumulation, leading to upregulation of interferon-stimulated genes and increased T cell cytotoxicity. In syngeneic tumour models, STC-15 induces durable antitumour immunity and synergizes with PD-1 blockade to achieve complete tumour regression. The completed Phase 1 trial (NCT05584111) established its safety and pharmacologic activity, while an ongoing Phase 1b/2 trial (NCT06975293) is evaluating STC-15 in combination with the PD-1 antibody toripalimab across advanced cancers—including non-small-cell lung carcinoma, melanoma, endometrial carcinoma, and head and neck squamous cell carcinoma—to define optimal dosing, safety, and T cell-modulating efficacy of METTL3 blockade. These findings underscore that manipulating RNA methylation can remodel the tumour-immune interface by enhancing antigen presentation, T cell infiltration, and effector persistence, positioning epitranscriptomic modulation as a viable adjunct to checkpoint therapy.

Beyond METTL3 inhibition, the functional tuning of m^6^A reader proteins also offers therapeutic promise. Loss of YTHDF2 enhances Th9 differentiation by stabilizing *Gata3* and *Smad3* mRNAs under IL-4 and TGF-β signalling, promoting IL-9 and IL-21 production, increased CD8^+^ T cell and NK cell infiltration, and improved antitumour efficacy. In CAR-Th9 cells, YTHDF2 depletion sustains immune activation and augments cytotoxic potential, identifying YTHDF2 as a negative epitranscriptomic checkpoint restraining T cell-mediated immunity 145.

Nevertheless, the dual identity of RNA-modifying enzymes complicates their therapeutic targeting. The same factor can exert oncogenic or tumour-suppressive effects depending on cellular context—for example, METTL3 deletion in melanoma cells suppresses tumour progression and enhances CD8^+^ T cell infiltration 20, whereas METTL3 loss in macrophages promotes tumour growth and impairs PD-1 blockade efficacy 146. Such context-dependent outcomes highlight the necessity for cell-type-specific delivery systems (e.g., lipid nanoparticles or virus-like particles) and temporal control mechanisms, including drug-inducible degrons or switchable RNA editors, to achieve precise and reversible modulation during T cell manufacturing or post-infusion phases.

Because RNA modifications are broadly involved in essential physiological processes, selective targeting within the tumour microenvironment without perturbing normal homeostasis remains a major challenge. Although advances in structure-guided drug design have yielded molecules with improved specificity, complete separation of therapeutic and physiological effects has not yet been achieved. The integration of standardized detection frameworks, cell-type-restricted delivery, and temporal control strategies will be key to transforming RNA modifications from static biomarkers into actionable determinants of immune behavior. Collectively, these advances establish a conceptual foundation for precision epitranscriptomic immunotherapy, in which RNA-modification pathways are harnessed to reprogram T cell metabolism, activation, and persistence in cancer and beyond.

## Conclusion

RNA modifications have emerged as a fundamental epitranscriptomic mechanism that integrates transcriptional, metabolic, and post-transcriptional regulation to orchestrate T cell activation, differentiation, and functional plasticity. Through the coordinated interplay of methyltransferases, demethylases, and reader proteins, these chemical modifications remodel the transcriptomic landscape and control RNA metabolism at multiple levels, coupling extracellular cues and intracellular metabolic states to precise gene-expression programs. This multilayered regulatory system enables T cells to balance effector expansion with immune tolerance, thereby maintaining immune homeostasis while preserving responsiveness to antigenic stimulation.

Across distinct T cell lineages, RNA modifications act as key determinants of lineage commitment, effector specification, and memory persistence. By modulating transcript stability, translational efficiency, and RNA decay, they synchronize transcriptional output with the biosynthetic and energetic demands of immune activation. Functioning as molecular rheostats, these modifications translate transient signaling inputs into durable transcriptional states that define activation thresholds, clonal fitness, and survival potential. Such epitranscriptomic regulation operates in concert with transcriptional and epigenetic programs, establishing a hierarchical control network that safeguards immune precision and adaptability.

The primary objective of this review is to delineate the conceptual and mechanistic framework through which RNA modifications govern T cell fate and function. By synthesizing recent advances across molecular, cellular, and pathological contexts, this work consolidates current understanding of how distinct RNA modifications converge to regulate transcript stability, translational control, and metabolic reprogramming, thereby shaping T cell behavior in immunity and disease. The rationale underlying this synthesis is to interpret the epitranscriptome as a dynamic and programmable interface that integrates signaling intensity, metabolic flux, and chromatin architecture into coherent immune outcomes. Through this perspective, RNA modification machinery emerges as a set of tunable molecular nodes with substantial therapeutic potential for the modulation of immune responses.

Despite these advances, several conceptual and technical challenges constrain current progress. Most available studies examine individual RNA modifications or single regulatory enzymes in isolation, providing limited insight into their combinatorial interactions or site-specific cross-regulation within the same transcript. The temporal and spatial dynamics of RNA modifications throughout T cell activation, differentiation, and memory maintenance remain insufficiently resolved. Furthermore, the interplay between RNA modifications and other epigenetic layers—such as DNA methylation, histone modifications, and chromatin accessibility—remains largely unexplored, despite their likely role in establishing integrated regulatory feedback. Methodological limitations in quantitative mapping, stoichiometric resolution, and single-cell detection further restrict mechanistic dissection. Translational application also requires the development of selective, reversible modulators of RNA-modifying enzymes and effective strategies for *in vivo* delivery, which remain in early stages of development.

Future research should aim to construct an integrative and spatiotemporally resolved map of the T cell epitranscriptome. The integration of single-cell multi-omics, spatial transcriptomics, and metabolic tracing will be instrumental in defining the dynamic coordination between RNA modifications, transcriptional programs, and metabolic adaptation. Advances in programmable RNA-modification editing and synthetic epitranscriptomic technologies may allow precise reconfiguration of T cell states, enabling targeted modulation of activation, persistence, and exhaustion. Ultimately, elucidating how RNA modifications encode, stabilize, and reprogram immune identity will provide the conceptual foundation for next-generation immunotherapeutic strategies based on epitranscriptomic modulation.

In summary, RNA modifications constitute a central regulatory axis that confers flexibility, precision, and endurance upon T cell immunity. They form the molecular syntax through which immune cells integrate signaling and metabolic information into defined transcriptional and functional states. Deciphering this epitranscriptomic code will not only advance our fundamental understanding of T cell biology but also open translational avenues for rationally engineering immune responses in infection, autoimmunity, and cancer immunotherapy.

## Figures and Tables

**Table 1:** Mechanisms of T Cell Regulation by RNA Modifications

T cell subset	Regulator	Modification	Target	Mechanism	Functional Outcome	Ref.
CD8^+^ T cells	METTL3	m^6^A writer	*Tbx21* (T-bet) mRNA	Enhancing mRNA stability and translation	Promoting effector differentiation and cytokine production	17
	FTO/IGF2BP3	m^6^A eraser/reader	*Fas* mRNA	FTO: Preventing excessive stabilization and translation of *Fas* mRNA; IGF2BP3: Stabilizing *Fas* mRNA and promoting its translation upon FTO loss	Preventing excessive apoptosis, maintaining CD8^+^ T cell survival and effector function	79
CD4^+^ T cells	YTHDF2	m^6^A reader	*TNF* mRNA	Binding m^6^A-modified transcripts and promoting their decay	Limiting TNF production to fine-tune inflammatory responses	80
	METTL3/FTO/YTHDF2	m^6^A writer/eraser/reader	*CD40L* mRNA	METTL3: Dynamically regulating mRNA stability; FTO/YTHDF2: Fine-tuning mRNA stability	Controlling T cell activation thresholds and cytokine production	85
	METTL3	m^6^A writer	*Tcf7* (TCF-1) mRNA	Stabilizing *Tcf7* mRNA and defining T_fh_ identity	Promoting T_fh_ cell differentiation and germinal center formation	86
	METTL14	m^6^A writer	*Foxp3* mRNA	Maintaining *Foxp3* mRNA stability and restraining mTOR-p70S6K signaling	Preserving iTreg suppressive function and preventing inflammation	87
γδ T cells	ALKBH5	m^6^A eraser	*Jagged1/Notch2* mRNA	Suppressing Notch signaling	Expanding the γδ T cell pool and enhancing mucosal defense	90
	METTL3	m^6^A writer	*Stat1* mRNA	Promoting *Stat1* mRNA decay and limiting dsRNA accumulation	Preserving γδT17 cell differentiation and IL-17 production	91
iNKT cells	METTL14	m^6^A writer	*Cish* mRNA	Regulating IL-2/IL-15 responsiveness and Vα14-Jα18 rearrangement	Supporting iNKT cell survival, maturation, and cytokine production	92
	METTL3	m^6^A writer	*Creb1* transcripts	Ensuring sufficient CREB1 protein abundance and phosphorylation	Maintaining iNKT lineage homeostasis, functional specialization and antitumor immunity	93
CD4^+^ T cells	NSUN2	m^5^C writer	*IL-17A* mRNA (C466 site)	Enhancing translational efficiency without altering mRNA abundance	Promoting IL-17A expression in Th17 cells	94
T cells	PUS10	Ψ modification	tRNA	Preventing tRNA fragmentation and inhibiting retrotransposon expression to avoid cGAS-STING pathway activation	Maintaining innate immune homeostasis and preventing autoimmunity	96
CD4^+^ T cells	TRMT61A/ TRMT6	m^1^A^58^ writer	Early-expressed tRNA subset	Enhancing translation of *MYC* and key effector proteins	Promoting cell cycle entry and clonal expansion	10
	RNMT	m^7^G writer	TOP mRNAs and snoRNAs	Promoting translation via ribosome biogenesis	Enabling proliferation and effector differentiation	98

**Table 2:** RNA modification-driven remodeling of T cell immunity in disease

Disease	RNA modifier	Molecular target	Immunological outcome	Implication	Ref.
Lung cancer	YTHDF2	Epi-transcriptional and transcriptional networks	Promoting T cell polyfunctionality	Potentiating T cell immunity	18
Oral squamous cell carcinoma	PCIF1	Ferroptosis-inhibitory gene	CD8^+^ T cell activation↑	Boosting anti-PD-1 immunotherapy	105
HCC	WTAP	Promoting PD1 expression	Inhibiting CD8^+^ T cells proliferation	Promoting HCC progression	132
NPC	/	/	CD8^+^ T cells and CD4^+^ T cells↑	Immune dysregulation	107
SLE	METTL3	Stabilizing *Foxp3* mRNA	CD4^+^ T cell activation↓	Suppressing Treg cell differentiation	109
SLE	MT-ND6	Increasing ROS and ATP	Inflammatory CD4^+^ T cells↓	Exhibiting mitochondrial dysfunction	110
SLE	NSUN2	/	Altering mRNA methylation patterns	Immune and inflammatory dysregulation	111
Colitis	NSUN2	Chromatin regions	Inhibiting Th17 cell differentiation	Enhancing mRNA stability	117
SLE	NAT10	/	Reducing ac^4^C levels	Modulating mRNA stability and translational initiation	112
Psoriasis	ALKBH5	Enhancing stability of *IL-17A* mRNA	Increasing IL-17A expression in CD4^+^ T cells	Promoting psoriasis-like phenotype and inflammation	113
GO	WTAP	promoting m^6^A deposition on *THBS1* transcripts	Promoting glycolysis of CD4^+^ T cells	Restoring T cell balance	114
Uveitis	METTL3	Enhancing stability in YTHDC2-dependent manner	Decreasing IL-17 and IL-23 receptor expression	Reducing pathogenic Th17 responses	115
IBD	NAT10	Diminishing Bag3 stability	Accelerating T cell apoptosis	Preserving T cell balance	116
Kidney transplantation	WTAP	Upregulation of Foxo1	Naïve T cells↓	Promoting Treg differentiation and function	118
Allograft rejection	METTL3	Decreasing in m^6^A levels	Reduction TEa cell proliferation	Suppressing alloreactive CD4^+^ T cell effector function and differentiation	119
Bacterial infection	FTO	/	CD4^+^ T cell differentiation↑	Decreasing T-bet and IFN-γ expression	122
EAE	METTL3	Stabilizing *SOCS3* mRNA	Disrupting Th17 pathogenic programs	Repressing IL-17A and CCR5 expression	125
Asthma	METTL3	Suppressing pri-miR-192-5p processing	Th1 cells↓	Driving to lipid metabolic reprogramming	127
IDC CAD	BRCA1	m^6^A RNA methylation alterations	CTL mediated cytotoxicity↑	Regulating tumor suppressor gene P53	129

## Abbreviations

ac^4^C: N^4^-acetylcytidine
CAD: coronary artery disease
CAR: chimeric antigen receptor
CNS: central nervous system
dsRNA: double-stranded RNA
EAE: experimental autoimmune encephalomyelitis
EMT: epithelial-mesenchymal transition
GO: Graves' ophthalmopathy
HCC: hepatocellular carcinoma
IBD: inflammatory bowel disease
IDC: invasive ductal carcinoma
iNKT: invariant natural killer T
iTregs: induced regulatory T cells
LUSC: lung squamous cell carcinoma
m^1^A: N^1^-methyladenosine
m^5^C: 5-methylcytosine
m^6^A: N^6^-methyladenosine
m^7^G: N^7^-methylguanosine
MT-ND6: mitochondrial-encoded NADH dehydrogenase 6
NPC: nasopharyngeal carcinoma
SCD1: stearoyl-CoA desaturase 1
SLE: Systemic lupus erythematosus
T_fh_: T follicular helper
THBS1: thrombospondin 1
Treg: regulatory T cell
Ψ: pseudouridine
